# Clinicopathological and molecular profiles of *Babesia vogeli* infection and *Ehrlichia canis* coinfection

**DOI:** 10.14202/vetworld.2020.1294-1302

**Published:** 2020-07-09

**Authors:** Thanakorn Rawangchue, Sivapong Sungpradit

**Affiliations:** 1 Parasitology Unit, Center for Veterinary Diagnosis, Faculty of Veterinary Science, Mahidol University, Nakhon Pathom, Thailand; 2 Department of Pre-Clinic and Applied Animal Science, Faculty of Veterinary Science, Mahidol University, Nakhon Pathom, Thailand

**Keywords:** 18S rRNA gene, *Babesia vogeli*, coinfection, *Ehrlichia canis*, hematobiochemical data, red blood cell

## Abstract

**Background and Aim:**

Canine babesiosis, a tick-borne parasitic disease, is caused by the hemoprotozoa, *Babesia vogeli*, and *Babesia gibsoni*. Infection with these parasites, which is endemic globally, leads to life-threatening immunosuppression in dogs. The merozoites invade the red blood cells (RBCs) of infected dogs. *Ehrlichia canis*, an intracellular bacterium that infects monocytes, is transmitted by the same tick species (*Rhipicephalus sanguineus*) during blood consumption and coinfection with *B. vogeli* and *E. canis* has been reported. Although the hematology and biochemistry of canine babesiosis have been studied, more studies are needed to develop a better understanding of the hematobiochemical and molecular profiles associated with cases of single infection and coinfection of canine babesiosis in Thailand. This study aimed to investigate the hematological, biochemical, and molecular profiles of *B. vogeli* infection and *E. canis* coinfection.

**Materials and Methods:**

The study included 33 *B. vogeli*–positive blood samples and 11 *E. canis*–coinfected blood samples. To exclude coinfection with *Hepatozoon canis* and *Anaplasma platys*, only dogs with *B. vogeli* infection and *B. vogeli*–*E. canis* coinfection were included in the study. A multiplex polymerase chain reaction (PCR) assay was conducted to detect *B. vogeli*, *E. canis*, and *H. canis*, and a conventional PCR assay was conducted for the detection of *A. platys*. Besides, the PCR assay and sequencing, comprehensive data analysis was conducted, including a microscopic blood parasite examination and hematological and biochemical data analysis.

**Results:**

The comparison of the hematobiochemical data between the *B. vogeli*–positive and *E. canis* coinfection groups identified that there were statistically significant differences in the RBC parameters, including RBC count, hemoglobin concentration, hematocrit, and RBC distribution width (p=0.001). Neither *B. vogeli* infection nor coinfection with *E. canis* was associated with the sex, breed, recorded clinical signs, geographic origin of the dog and also *B. vogel*i 18S rRNA gene sequencing results.

**Conclusion:**

Coinfection with *E. canis* increased the severity of babesiosis. The pathogenic mechanisms underlying this infection, such as destruction of RBCs, require further investigation. This study may enhance diagnosis, treatment, and prevention of canine babesiosis.

## Introduction

Canine babesiosis, which occurs worldwide, is a tick-borne disease caused by the small (*Babesia gibsoni*, *Babesia conradae*, and *Babesia vulpes*) and large (*Babesia vogeli*, *Babesia canis*, and *Babesia rossi*) *Babesia* groups [1-3] and is a life-threatening condition for dogs [4]. The previous studies have reported the sizes of the small and large intraerythrocytic-stage merozoites as measuring 1×3 μm and 2-2.5×4.5-5 μm in size, respectively [5,6]. The trophozoite and merozoite morphology in red blood cells (RBCs) is typically ring-shaped and forms pyriform bodies, respectively [7]. The main species of canine babesiosis in Thailand is *B. vogeli*, carried by *Rhipicephalus sanguineus*, which is commonly known as the brown dog tick [8-12].

*Babesia*-infected dogs may be asymptomatic [8] or present with various clinical signs that range from mild to peracute and deadly. Clinical manifestations include anorexia, lethargy, fever, pale mucous membrane, jaundice, and renal disease [13-15], depending on the parasite and the host’s, age, sex, and breed [12,16]. Natural and experimental *in vivo* infections with *B. vogeli* have shown subclinical signs. In immunosuppressed animals, such as splenectomized dogs, the pathogen causes severe acute infection, which is followed by fever, anorexia, malaise, regenerative anemia, thrombocytopenia, and increased white blood cell (WBC) count [6,17]. By contrast, many studies have reported that there are decreased numbers of WBC in dogs with canine babesiosis [18,19]. A retrospective canine babesiosis study in the Small Animal Teaching Hospital of Chulalongkorn University in Thailand found that hypocytic hypochromic anemia and thrombocytopenia were the major clinical hematological findings in dogs with canine babesiosis [20]. However, other studies have shown the occurrence of macrocytic hyperchromic anemia in canine babesiosis [18,19]. Hematological profiles of canines with coinfections of *Babesia*–*Ehrlichia* have been studied in South Africa, but the prevalence of coinfection in South Africa is low and the main hemoprotozoan species is *B. rossi* [21]. Although coinfections have frequently been reported in Thailand in the literature describing the canine hemoparasite prevalence [9,10,22], information about the hematobiochemical patterns of the infection and the molecular diversity of *B. vogeli* and *Ehrlichia canis* coinfection remains lacking.

This study aimed to investigate 44 *Babesia-*positive blood samples, including samples that had a single infection with *Babesia* and samples that had *Babesia*–*Ehrlichia* concurrent infections that were confirmed by microscopic and molecular examinations. Comprehensive and hematobiochemical data were analyzed, and *Babesia* 18S rRNA and *Ehrlichia* 16S rRNA genes were sequenced for the identification of *Babesia* subspecies, as well as for the genetic variation of both hemoparasites.

## Materials and Methods

### Ethical approval

The study protocol (no. MUVS-2018-12-66) was approved by the Faculty of Veterinary Science-Animal Care and Use Committee (FVS-ACUC), Mahidol University, Thailand.

### Comprehensive data collection

**Figure-1:**
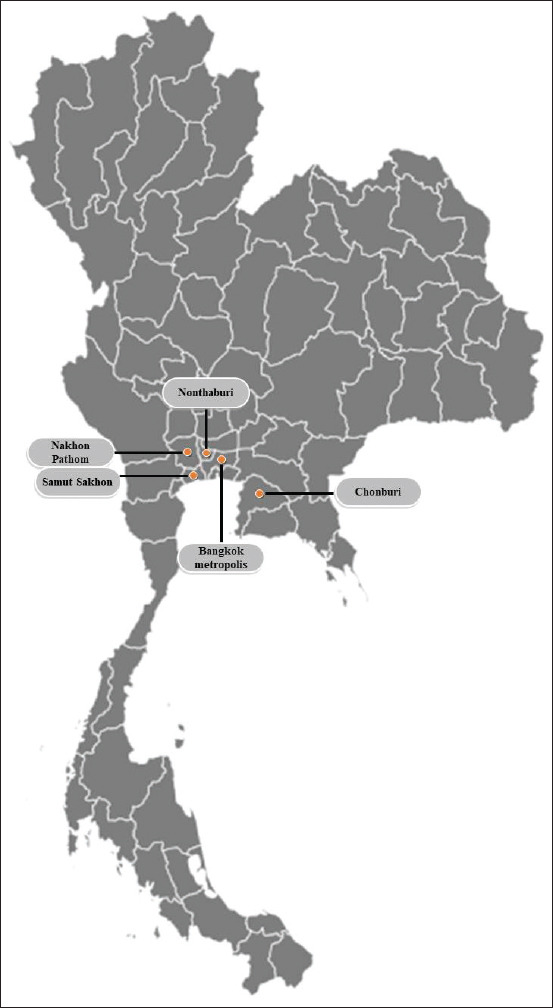
Geographic distribution of the babesiosis cases in this study.

Retrospective comprehensive data, including signalment (age, sex, breed, and geographic origin), recorded clinical signs, and hematobiochemical profiles, from 44 dogs were gathered from medical records provided by Prasu-Arthorn Animal Teaching Hospital, Mahidol University, on the 1^st^ day of registration, between February and December 2019. A map showing the provinces in Thailand where the babesiosis cases were located was drawn using a template obtained from www.simplemaps.com (Figure-1).

### Blood sample preparation

The blood samples (1 mL) were obtained from Prasu-Arthorn Animal Teaching Hospital, Faculty of Veterinary Science, Mahidol University, Salaya Campus, Nakhon Pathom, Thailand. The samples were drawn from the cephalic or saphenous veins of the dogs and preserved in ethylenediaminetetraacetic acid in plain tubes for hematobiochemical analysis. Thin buffy coat smears were prepared and stained using Giemsa solution and sent to the laboratory of the parasitology unit for routine microscopic detection. Thirty-three *Babesia–* positive samples and 11 samples that had *Babesia*–*Ehrlichia* coinfection were identified and further confirmed by molecular examination.

### DNA preparation

DNA was isolated from 200 μL of blood from samples positive for *Babesia* and *Babesia*–*Ehrlichia* coinfection using a NucleoSpin Blood Kit (Macherey-Nagel GmbH & Co. KG, Düren, Germany), according to the manufacturer’s instructions. Briefly, 200 μL of blood sample were incubated with 25 μL proteinase K and 200 μL of binding buffer at 70°C for 15 min. DNA was eluted in 50 μL of elution buffer preheated at 70°C. It was then stored at −20°C until use.

### *B. vogeli* and *B. vogeli–E. canis* coinfection 18S rRNA gene amplification and sequencing

DNA isolated from blood samples was used as a template to amplify the majority of the 18S rRNA genes of *Babesia* spp. using multiplex polymerase chain reaction (PCR) [23] to verify *B. vogeli* and *E. canis* and exclude *Hepatozoon canis* infections in the samples.

The multiplex PCR amplifications were conducted using 5 μL of genomic DNA with the primers Ba103F, Ba721R, Ehr1401F, Ehr1780R, Hep001F, and Hep737R. The PCR reactions contained 5 μL total DNA template, 20 μL of the reagents which composed of 0.4 pmol of each primer, and 300 μM of each deoxyribonucleotide triphosphate (dNTP), four units of HotSta*rTaq* DNA Polymerase (QIAGEN, Hilden, Germany), 1X PCR buffer (20 mM Tris–HCl pH 8.4, 50 mM KCl), 1.5 mM MgCl_2_, and RNase-free water to a total volume of 25 μL. The reactions were conducted in a S1000 Thermal Cycler (Bio-Rad, Hercules, CA, USA), with the following steps: 15 min at 95°C; 30 cycles of 45 s at 94°C, 45 s at 65°C, and 90 s at 72°C; and 10 min at 72°C. The PCR products were examined through gel electrophoresis on a 2.5% agarose gel stained with GelRed (Biotium, Hayward, CA, USA) and visualized under ultraviolet (UV) light (GeneGenius, Cambridge, UK). The amplicons (619 base pairs) were then purified using QIAquick PCR purification kits (QIAGEN Inc., Valencia, CA, USA), according to the manufacturer’s protocol. Bidirectional sequencing of all PCR products was conducted using First BASE Laboratories (Selangor, Malaysia). The DNA sequences were then analyzed using an ABI 3730*xl* sequencer (Applied Biosystems, Foster City, CA, USA) with fluorescent dye terminator sequencing. The DNA sequencing was conducted using the *Babesia*-specific primers Ba103F and Ba721R.

### *Anaplasma platys* conventional PCR

Conventional PCR for the detection of *A. platys* was used to exclude *A. platys* coinfection. The reactions were conducted using 5 μL of the total DNA as a template and 20 μL of 0.4 pmol of each primer (Ana45F: 5′GTCGAACGGATTTTTGTCGT3′ and Ana671R: 5′GCCACTGGTGTTCCTCCTAA3′) [24], 300 μM of each dNTP, four units of *iTaq* DNA Polymerase (iNtRON Biotechnology, Kyungki-Do, South Korea), 1X PCR buffer (20 mM Tris–HCl pH 8.4, 50 mM KCl), 1.5 mM MgCl_2_, and RNase-free water. The amplification was conducted in a T100 Thermal Cycler (Bio-Rad, Hercules, CA, USA). The thermocycling steps consisted of one step for 10 min at 95°C followed by 35 cycles of 30 s at 94°C, 30 s at 55°C, and 45 s at 72°C, with a final extension step of 10 min at 72°C. Aliquots of the amplicons were detected using gel electrophoresis on 2.0% agarose gel stained with GelRed (Biotium, Hayward, CA, USA) and visualized under UV light (Syngene, Cambridge, UK).

### *E. canis* 16S rRNA gene amplification and sequencing

For the detection of *E. canis*, conventional PCR was used to amplify the 16S rRNA genes. The reactions were conducted using 5 μL of the total DNA template and 20 μL of 0.4 pmol of each primer (Ecan 16S-94F: 5′GTGGCAGACGGGTGAGTAAT3′ and Ecan 16S-1102R: 5′GAGTGCCCAGCATTACCTGT3′), 300 μM of each dNTP, four units of *iTaq* DNA Polymerase (iNtRON Biotechnology, Kyungki-Do, South Korea), 1X PCR buffer (20 mM Tris–HCl pH 8.4, 50 mM KCl), 1.5 mM MgCl_2_, and RNase-free water. The amplification was conducted in a T100 Thermal Cycler (Bio-Rad, Hercules, CA, USA). The thermocycling consisted of one step of 10 min at 95°C followed by 35 cycles of 30 s at 94°C, 30 s at 58°C, and 45 s at 72°C, with a final extension step of 10 min at 72°C. Aliquots of the amplicons were detected using gel electrophoresis with a 2.0% agarose gel stained with GelRed (Biotium, Hayward, CA, USA) and visualized under a UV light (Syngene, Cambridge, UK). The amplicons (1009 base pairs) were then purified using the QIAquick PCR purification kit (QIAGEN Inc., Valencia, CA, USA), according to the manufacturer’s protocol. Bidirectional sequencing of all PCR products was conducted using First BASE Laboratories (Selangor, Malaysia). The DNA sequences were then analyzed using an ABI 3730*xl* sequencer (Applied Biosystems, Foster City, CA, USA) with fluorescent dye terminator sequencing. The DNA sequencing was conducted using *E. canis-* specific primers Ecan 16S-94F and Ecan 16S-1102R.

### Bioinformatic and phylogenetic analysis

The *B. vogeli* 18S rRNA and *E. canis* 16S rRNA sequence results were analyzed using several programs. All sequencing results were compared with sequences available in the GenBank database using the Basic Local Alignment Search Tool (http://blast.ncbi.nlm.nih.gov/Blast.cgi). Multiple alignments of all nucleotide sequences were conducted using the ClustalW web-based tool (https://www.genome.jp/tools-bin/clustalw) [25]. Phylogenetic trees were reconstructed using maximum likelihood analysis with bootstrapping (100 replications) in the advanced mode of the Phylogeny.fr web server (http://www.phylogene.fr/) [26]. All sequences were compared with published sequences in the GenBank database that originated from other geographic locations globally. *H. canis* and *Trypanosoma evansi* were included as the outgroup for the 18S rRNA and *A. platys* was included as the outgroup for the 16S rRNA phylogenetic tree, respectively.

### Statistical analysis

The commercial software IBM SPSS Statistics (IBM, Armonk, NY, USA) version 18 was used for data analysis. Independent *t*-tests were used to compare the sexes and breeds of the *B. vogeli*–infected group with the coinfected group. The clinical signs and geographic origins were evaluated using one-way analysis of variance. The ages and hematobiochemical data of the infected dogs were compared using the Mann–Whitney U*-*test. p<0.05 was considered statistically significant.

## Results

### Analysis of comprehensive data

The 44 dog samples were naturally infected with *B*. *vogeli* or coinfected with *B*. *vogeli* and *E. canis*. The microscopic screening and conventional and multiplex PCR results confirmed either the single *B. vogeli* infection (33 samples, 75%) or coinfection with *E. canis* (11 samples, 25%).

The median age of the 33 Babesia-infected dogs was 36 months, with a range from 1 to 156 months. This group included 19 (57.60%) male and 14 (42.40%) female dogs (Table-1). The most prevalent breeds were mixed breeds (15/33=45.45%) and Poodles (5/33=15.15%), and the third most prevalent breeds were Shih Tzus, Siberian Huskies, and Cocker Spaniels (2/33=6.06%). The most common clinical sign was anorexia with or without depression or fever (13/33=39.40%) (Table-1). The main geographic origin of the coinfected dogs was the Bangkok Metropolis (16/33=48.49%), followed by Nakhon Pathom (11/33=33.33%), Nonthaburi (5/33=15.15%), and Chonburi (1/33=3.03%) provinces (Figure-1 and Table-1).

**Table-1:** Comparison of sex, breed, age, clinical signs, and geographic origin of the *B. vogeli* infection and *E. canis* coinfection cases.

Parameters	n (%)	Median (range)	Statistic type	p-value
Sex		N/A	Independent t-test	0.79
*B. vogeli* infection cases	33			
Male	19 (57.60)			
Female	14 (42.40)			
*E. canis* coinfection cases	11			
Male	7 (63.64)			
Female	4 (36.36)			
Breed		N/A	Independent t-test	0.48
*B. vogeli* infection cases	33			
Mixed breed	15 (45.45)			
Pure breed	18 (54.55)			
*E. canis* coinfection cases	11			
Mixed breed	7 (63.64)			
Pure breed	4 (36.36)			
Age (months)			Mann-Whitney *U* test	0.57
*B. vogeli* infection cases	33	36 (1-156)		
*E. canis* coinfection cases	11	71 (2-120)		
Clinical sign		N/A	One-way ANOVA	0.57
*B. vogeli* infection cases	33			
Anorexia	13 (39.40)			
Musculoskeletal disorder	6 (18.18)			
Others	14 (42.42)			
*E. canis* coinfection cases	11			
Anorexia	5 (45.46)			
Musculoskeletal disorder	2 (18.18)			
Others	4 (36.36)			
Geographic origin		N/A	One-way ANOVA	0.89
*B. vogeli* infection cases	33			
Bangkok metropolis	16 (48.49)			
Nakhon Pathom	11 (33.33)			
Nonthaburi	5 (15.15)			
Chonburi	1 (3.03)			
*E. canis* coinfection cases	11			
Bangkok metropolis	6 (54.55)			
Nakhon Pathom	3 (27.27)			
Samut sakhon	2 (18.18)			

B. vogeli=Babesia vogeli, E. canis=Ehrlichia canis

The median age of the 11 Babesia–*Ehrlichia* coinfected dogs was 71 months, with a range from 2 to 120 months. This group included seven (63.64%) male and four (36.36%) female dogs. The most prevalent breed was mixed breed (7/11=63.64%), followed by Shih Tzus and Pomeranians (2/11=18.18% each). The most common clinical sign was anorexia with or without depression (5/11=45.46%). Other clinical signs observed in the remaining dogs (4/11=36.36%) included fever, abdominal distention, uveitis, and hypoglycemia. The major geographic origin of the coinfected dogs was the Bangkok Metropolis (6/11=54.55%), followed by Nakhon Pathom (3/11=27.27%), and Samut Sakhon (2/11=18.18%) provinces (Figure-1 and Table-1).

As shown in Table-1, the sex (male and female), breed (pure and mixed), age, clinical signs, and geographic origin of the dogs were not significantly different between the *B. vogeli* and the coinfected group (p=0.79, 0.48, 0.57, 0.57, and 0.89, respectively).

### Analysis of hematobiochemical data

The RBC count, hemoglobin concentration, hematocrit, and RBC distribution width (RDW) were significantly different between the *B. vogeli* and the coinfected group (p=0.001, 0.001, 0.001, and 0.005, respectively). The RBC count, hemoglobin concentration, and hematocrit levels in the coinfected group were lower than the reference value ranges, whereas the RDW was higher. The platelet count was not significantly different (p=0.125) between the groups. Both groups exhibited thrombocytopenia (a platelet count of less than 200×10^3^ cells/μL), with 32/33 (96.97%) dogs in the *B. vogeli-*infected group and 9/11 (81.82%) dogs in the coinfected group affected. The WBC count was not significantly different between the two groups (p=0.86). Differential counting of the WBCs showed that there was significantly different lymphocytosis (p=0.013) in the *B. vogeli-*infected group (Table-2).

**Table-2:** Comparison of hematological and blood chemical profiles of *B. vogeli* infection and *E. canis* coinfection cases. Statistical differences were assessed by the Mann–Whitney U-test. Statistically significant differences are marked with an asterisk.

Parameters	n	Median (range)	References (range)	p-value
Hematology				
Hemoglobin concentration (g/dL)			10-18	0.001*
*B. vogeli* infection cases	33	11.8 (3.5-17.7)		
*E. canis* coinfection cases	11	6.6 (2.0-11.8)		
Red blood cell count (RBC)×10^6^ (cells/mm^3^)			5-9	0.001*
*B. vogeli* infection cases	33	4.96 (1.60-7.15)		
*E. canis* coinfection cases	11	3.00 (1.00-4.69)		
hematocrit (%)			35-55	0.001*
*B. vogeli* infection cases	33	35.0 (10.4-50.3)		
*E. canis* coinfection cases	11	20.1 (5.5-32.8)		
Mean cell volume (fL)			60-77	0.242
*B. vogeli* infection cases	33	69 (59-92)		
*E. canis* coinfection cases	11	67 (59-74)		
Mean corpuscular hemoglobin (pg)			20-25	0.242
*B. vogeli* infection cases	33	23.5 (20.0-32.8)		
*E. canis* coinfection cases	11	22.4 (17.4-26.5)		
Mean corpuscular hemoglobin concentration (g/dL)			32-36	0.724
*B. vogeli* infection cases	33	34.0 (30.0-35.8)		
*E. canis* coinfection cases	11	34.0 (29.4-36.3)		
RBC distribution width (%)			12-15	0.005*
*B. vogeli* infection cases	33	15.2 (13.9-19.5)		
*E. canis* coinfection cases	11	16.9 (14.9-19.7)		
White blood cell count (cells/μL)			6000-17000	0.86
*B. vogeli* infection cases	33	7700 (3900-39000)		
*E. canis* coinfection cases	11	11300 (1500-25200)		
Segmented neutrophils (cells/μL)			2060-10600	0.818
*B. vogeli* infection cases	33	6364 (560-35880)		
*E. canis* coinfection cases	11	7973 (0-24192)		
Band neutrophils (cells/μL)			0-300	0.081
*B. vogeli* infection cases	33	0 (0-98)		
*E. canis* coinfection cases	11	0 (0-84)		
Monocytes (cells/μL)			0-840	0.681
*B. vogeli* infection cases	33	188 (0-3500)		
*E. canis* coinfection cases	11	228 (0-1470)		
Lymphocytes (cells/μL)			0-840	0.013*
*B. vogeli* infection cases	33	1470 (196-7616)		
*E. canis* coinfection cases	11	567 (0-3332)		
Eosinophils (cells/μL)			0-840	0.087
*B. vogeli* infection cases	33	0 (0-1064)		
*E. canis* coinfection cases	11	0 (0-595)		
Basophils (cells/μL)			0-840	0.564
*B. vogeli* infection cases	33	0 (0-133)		
*E. canis* coinfection cases	11	0 (0-0)		
Platelet count×10^3^(cell/μL)			200-500	0.125
*B. vogeli* infection cases	33	41 (6-217)		
*E. canis* coinfection cases	11	43 (23-254)		
Plasma protein (g/L)			6.0-7.5	0.357
*B. vogeli* infection cases	32	9.0 (7.6-10.6)		
*E. canis* coinfection cases	11	8.2 (5.6-12.0)		
Biochemistry				
Alanine aminotransferase (ALT) (U/L)			0-50	0.51
*B. vogeli* infection cases	26	44.5 (5.0-358.0)		
*E. canis* coinfection cases	9	59.0 (13.0-133.0)		
Creatinine (mg/dL)			0.5-1.8	0.153
*B. vogeli* infection cases	28	0.9 (0.5-2.8)		
*E. canis* coinfection cases	9	1.5 (0.3-3.3)		

B. vogeli=Babesia vogeli, E. canis=Ehrlichia canis

Plasma protein was not significantly different (p=0.357), with both groups exhibiting hyperproteinemia (plasma protein more than 7.5 g/L). All 33 (100%) dogs in the *B. vogeli-*infected group and 8/11 (72.73%) dogs in the coinfected group displayed hyperproteinemia (Table-2).

### Phylogenetic tree analysis

**Figure-2:**
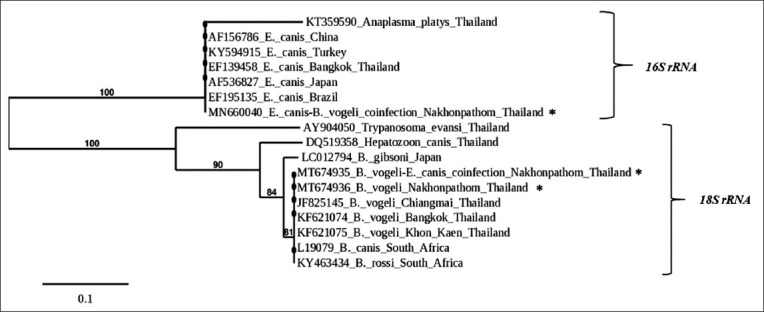
Phylogenetic tree analysis of *Babesia vogeli* 18S rRNA and *Ehrlichia canis* 16S rRNA gene sequences of dog samples from Nakhon Pathom, Thailand (asterisk), and other provinces and countries. Bootstrap values are provided at the beginning of each branch.

Phylogenetic analysis revealed that our sequences were closely related to the sequences from Thailand available in GenBank. The nucleotide sequences obtained from bidirectional sequencing of the 18S rRNA sequences of *B. vogeli* with coinfection of *E. canis* and the single *B. vogeli* infection groups (MT674935 and MT674936) showed that both nucleotide sequences were identical to those previously reported in Thailand (Chiangmai); *B. vogel*i 18S rRNA sequences (JF825145). The 16S rRNA sequences from *E. canis* in the coinfection group (MN660040) were identical to previously reported *E. canis* 16S rRNA sequences (EF139458) from samples from Thailand (Bangkok) (Figure-2).

## Discussion

*R. sanguineus* is a common hard tick in Thailand (Southeast Asia) that can carry various canine hemoparasites, including *E. canis*, *B. vogeli*, *H. canis*, *A. platys*, and *Mycoplasma* spp., which infect both domestic and stray dogs [12,22,27-29]. Occurrences of *B. vogel*i and *E. cani*s coinfection and sole *B. vogeli* infections confirmed by molecular examination have been reported in Thailand [9,22]. Studies on tick-borne hemoparasite prevalence found that the ratio of coinfections of *B. vogeli* and *E. canis* to single *B. vogeli* infections was 2:59 (1:29.5) in Khon Kaen Province [22] and 2:17 (1:8.5) in Mahasarakham Province [9]. In the present study, to have a reliable sample comparison, we gathered 11:33 (1:3) ratio of samples of these infections from small animal hospital cases. The dogs of both groups were mainly from the Bangkok Metropolis and bordering areas, such as the Nakhon Pathom, Samut Sakhon, and Nonthaburi provinces. By contrast, a study conducted in South Africa, where the single *Babesia* pathogen was *B. rossi*, found that the ratio was 4:191 (1:48) [21]. To the best of our knowledge, the present study is the first to investigate the hematobiochemical parameters of *B. vogeli* infections and *B. vogeli* and *E. canis* coinfections.

In a hematobiochemical study, Niwetpathomwat *et al*. [20] reported decreases in the hemoglobin concentration, mean corpuscular hemoglobin (MCH), MHC concentration (MCHC), and mean platelet counts in registered babesiosis cases in the Chulalongkorn University’s Small Animal Teaching Hospital in Bangkok. Moreover, a study investigating cases of *B. vogeli* and *E. canis* coinfection in Costa Rica identified that the main clinical signs of young coinfected dogs were anemia, lethargy, and fever [30]. A 3-month-old coinfected puppy in the Philippines had lack of appetite, a pale mucous membrane, and fever, with thrombocytopenia as the most important abnormal hematological finding [31]. However, these results are not consistent with the findings of our study. In our study, in the group infected with *B. vogeli*, the only similar result was a decrease in the platelet count; the hemoglobin concentration, median MCH, and median MCHC values were all within the reference ranges. However, the study by Niwetpathomwat *et al*. [20] did not confirm the single *B. vogeli* infection in the canine babesiosis samples by molecular examination. Importantly, in our investigation, the RBC count, hemoglobin concentration, and median hematocrit levels in the cases with coinfection were significantly lower than in the cases with single *B. vogeli* infection. The previous studies demonstrated that infections of *E. canis*, which were confirmed using molecular techniques, led to a significant reduction in RBC count and hematocrit levels [32]. This study also demonstrated that anemic dogs infected with *Mycoplasma* spp. that had hematocrit levels of less than 15% had a seven-fold risk of coinfection with *E. canis*, as compared with a single infection of *Mycoplasma* spp. [28]. Coinfection with *E. canis* may lead to increased infection severity.

The significant increase in RDW in our ­coinfection group may be associated with increased destruction of RBCs, which may lead to regenerative anemia, including immune-mediated hemolytic anemia. RDW is related to heterogeneous erythrocyte populations in the blood circulation, whose main population is reticulocytes, rather than mature RBCs [33]. The mechanism underlying canine babesiosis has been proposed to involve intravascular and extravascular hemolysis with immune-mediated hemolytic anemia [34]. Various types of anemia, such as normocytic normochromic anemia, caused by *B. canis* and *B. gibsoni* infections [35], and hypocytic hypochromic anemia, caused by *B. vogeli* infection [20], have been reported. Our results indicate that coinfection with *E. canis* may cause macrocytic and/or microcytic hypochromic anemia, leading to the destruction of erythrocytes through an immune-mediated mechanism [36-38] resulting in the elevation of RDW.

The significance of lymphocytosis in single *B. vogeli* infections observed in this investigation is consistent with the previous studies conducted in Egypt [39], Italy [40], and Indonesia [41], although one study in Thailand did not describe lymphocytosis [20].

Niwetpathomwat *et al*. [20] reported that the levels of alkaline phosphatase (ALP) enzyme increased in cases of babesiosis. In the present study, we did not measure the levels of ALP and aspartate aminotransferase (AST), a liver enzyme, which are related to infections with *B. canis* and *B. gibsoni* [3,42]. There are three main ALP isoenzymes in canine serum [43] and the elevation of canine ALP is also associated with hepatobiliary, hepatic, and bone diseases [44-46]. In azotemic dogs infected with *B. canis*, the AST/ALT ratio decreased [47], and this ratio was not significantly different from those observed in *B. vogeli* infections [12].

In our study, the 44 bidirectional sequences of *B. vogeli* 18S rRNA displayed 100% identity and showed conservation with the various *B. vogeli* 18S rRNAs available in the GenBank database, including those from Chiangmai Province (the northern part of Thailand, JF825145) [8], Bangkok Metropolis (the central part of Thailand, KF621061-KF621074), and Khon Kaen Province (the north-eastern part of Thailand, KF621075-KF621081) [48]. By contrast, the sequences (around 200 base pairs) obtained from the Songkhla Province (the southern part of Thailand, KU765196 and KU765197) [49] had various genetic variations when aligned with our data. The genetic variation and genotyping of *B. vogeli* in Thailand should be further investigated using an immunodominant protein gene [50] with high levels of nucleotide diversity, such as an apical membrane antigen 1 [51].

## Conclusion

Coinfection with *E. canis* increases the severity of babesiosis. Its pathogenic mechanisms, such as RBC destruction, should be further investigated. This study may contribute to improve the diagnosis, treatment, and prevention of the disease.

## Authors’ Contributions

TR collected the samples and data. SS responsible for data analysis. Both authors conducted laboratory tests, wrote and revised the manuscript, and approved the final manuscript for submission.
